# Advanced Flexible Wearable Electronics from Hybrid Nanocomposites Based on Cellulose Nanofibers, PEDOT:PSS and Reduced Graphene Oxide

**DOI:** 10.3390/polym16213035

**Published:** 2024-10-29

**Authors:** Ana Carrascosa, Jaime S. Sánchez, María Guadalupe Morán-Aguilar, Gemma Gabriel, Fabiola Vilaseca

**Affiliations:** 1 Polymer Materials and Composites, Department of Industrial and Materials Science, Chalmers University of Technology, SE-412 96 Gothenburg, Sweden; ana.carrascosa.galan@gmail.com; 2 Materials and Manufacture, Department of Industrial and Materials Science, Chalmers University of Technology, SE-412 96 Gothenburg, Sweden; jaime.sanchez@smoltek.com; 3 R&D Department, China Three Gorges (Europe) S.A., C. del Príncipe de Vergara, 112, Planta 7, 28002 Madrid, Spain; 4 Advanced Biomaterials and Nanotechnology, Department of Chemical and Agricultural Engineering, and Agrifood Technology, University of Girona, 17003 Girona, Spain; mariaguadalupe.moran@udg.edu; 5 Instituto de Microelectrónica de Barcelona, IMB-CNM (CSIC), Campus UAB, Bellaterra, 08193 Barcelona, Spain; gemma.gabriel@imb-cnm.csic.es; 6 CIBER de Bioingeniería, Biomateriales y Nanomedicina, Instituto de Salud Carlos III, 28029 Madrid, Spain; 7 Department of Fibre and Polymer Technology, Wallenberg Wood Science Centre, KTH Royal Institute of Technology, SE-100 44 Stockholm, Sweden

**Keywords:** cellulose nanofibers, reduced graphene oxide, PEDOT:PSS, 3D structure, flexible electrodes, wearable electronics, responsible electronics

## Abstract

The need for responsible electronics is leading to great interest in the development of new bio-based devices that are environmentally friendly. This work presents a simple and efficient process for the creation of conductive nanocomposites using renewable materials such as cellulose nanofibers (CNF) from enzymatic pretreatment, poly(3,4-ethylenedioxythiophene)-poly(styrenesulfonate) (PEDOT:PSS), and/or reduced graphene oxide (rGO). Different combinations of CNF, rGo, and PEDOT:PSS were considered to generate homogeneous binary and ternary nanocomposite formulations. These formulations were characterized through SEM, Raman spectroscopy, mechanical, electrical, and electrochemical analysis. The binary formulation containing 40 wt% of PEDOT:PSS resulted in nanocomposite formulations with tensile strength, Young’s modulus, and a conductivity of 70.39 MPa, 3.87 GPa, and 0.35 S/cm, respectively. The binary formulation with 15 wt% of rGO reached 86.19 MPa, 4.41 GPa, and 13.88 S/cm of the same respective properties. A synergy effect was observed for the ternary formulations between both conductive elements; these nanocomposite formulations reached 42.11 S/cm of conductivity and kept their strength as nanocomposites. The 3D design strategy provided a highly conductive network maintaining the structural integrity of CNF, which generated homogenous nanocomposites with rGO and PEDOT:PSS. These formulations can be considered as greatly promising for the next generation of low-cost, eco-friendly, and energy storage devices, such as batteries or electrochemical capacitors.

## 1. Introduction

The demand for accelerated technological development and constant global growth is making it necessary to develop the generation of new green energy devices. This will contribute to reducing the problems caused by the massive accumulation of electrical and electronic waste that cannot naturally degrade and causes environmental toxicity and greenhouse emissions [1]. Flexible electronic technologies such as portable and electronic devices are currently considered promising power sources [2]. Therefore, a variety of active materials (e.g., carbon nanotubes, polyaniline, polypyrrole, transition metal oxides, etc.) have been managed for the design and assembly of flexible supercapacitor electrodes. However, some of these nanomaterials cannot form mechanically stable independent films, and some of them do not contribute to the capacitance properties. Consequently, it is of great importance to develop independent electrodes with robust mechanical flexibility that are made from highly specific electrode material. This will enable the fabrication of high-performance flexible devices [3].

In this context, to minimize environmental impact and develop a more sustainable future, research has recently been carried out on low-cost, energy-efficient, carbon-based green materials; these materials are considered candidates to replace some non-conventional materials in the manufacture of electronic devices [4]. Among carbon-based green materials, the most important classes are made from natural resources, such as cellulose, starch, chitosan, casein, soy protein, and collagen. Cellulose is the most promising natural polymer; it has been recognized as a sustainable, renewable, inexpensive, and non-toxic biomass resource. In addition, cellulose is composed of a mass of nanoscale fibrils that are designated as nanocellulose materials. This demonstrates its mechanical strength, hydrophilic surface, abundant hierarchical structure, and its capacity for transportation of ions within electrolytes. Also, nanocellulose can be formed by cellulose nanofiber or cellulose nanocrystal [5,6]. 

Cellulose nanofibrils (CNFs) are semicrystalline and flexible, with a diameter of 100 nm or less and a length of 500 nm or longer. They also have superior mechanical strength, large specific surface area, a high aspect ratio, an adjustable surface chemistry, and biodegradability [7]. In addition, due to the presence of abundant hydroxyl groups on the surface and the semicrystalline structure, CNF shows an outstanding tendency to form mechanically stable nanostructures with different reinforcement fillers or polymers via grafting. Consequently, CNFs have emerged as promising nanomaterials with great application potential, including nanocomposites, functional films, hydrogels in biomedicine, aerogels and foams for structural insulating materials, and barrier membranes for packaging [8,9,10]. Therefore, CNFs are regarded as a great candidate to substitute petroleum-based materials for novel composites production and renewable energy devices that are low cost, environmentally friendly, and have tunable mechanical and electrochemical properties [11].

Nevertheless, the poor electrical conductivity of CNFs restricts their application in supercapacitors. Hence, CNF is usually combined with conductive polymers to obtain nanocomposites that can form electron channels and promote electrical conductivity. This allows developers to change their functionality from substrates to active components of the device [12]. Reduced graphene oxide (rGO) and poly (3,4-ethylenedioxythiphoenes)-polystyrene sulfonate (PEDOT:PSS) have attracted great attention as components that can be combined with CNF due to their higher conductivity, better stability in ambient conditions, and their easier processability in comparison with other conducting polymers [13]. 

Graphene oxide (GO) is performed by exfoliation of graphite oxide and reduced graphene oxide (rGO) comes from the reduction of GO [10]. Thus, rGO is another promising material, having high capacitance and extremely long cycle life owing to its ultrahigh specific surface area and the electric double-layer mechanism that is the basis of its charge-discharge process [14]. Likewise, the PEDOT:PSS is a conductive polymer with excellent solution processability and outstanding optical/electrical properties. In consequence, rGo and PEDOT:PSS has been used as electrode materials for supercapacitors, however, bulk films with suitable flexibility and mechanical stability are still challenging [15]. 

In order to obtain the desirable properties of efficient films, it is necessary to consider the filler dispersibility in the polymer, filler–matrix interactions, and the ratio of filler to matrix [10]. Consequently, this work presents the analysis of the morphology, and the mechanical, electrical, and electrochemical properties for the development of binary films composed of CNF and rGO or PEDOT:PSS studying different mass loadings as well as the ternary combination of these elements to produce a film with mechanical robustness and flexible properties, high specific capacitance and excellent cyclic stability for the fabrication of high-performance, biocompatible and non-toxic flexible electronic devices, which promote sustainable and renewable energy storage.

## 2. Experimental

### 2.1. Materials

The cellulosic raw material (CRM) was supplied by Nordic Paper Säffle AB (Säffle, Sweden) with a composition reported of 87% of pure cellulose, 13% of hemicelluloses, and <1% of lignin. 

The graphene oxide water dispersion 0.4 wt% was purchased from Graphenea (Donostia, Spain) and the poly (3,4-ethylenedioxythiophene)–poly(styrenesulfonate) (PEDOT:PSS) 3.00–4.00 wt% in water (Heraeus, Hanau, Germany) and hydroiodic acid 57 wt% from Sigma-Aldrich. Nitrocellulose membrane disk diam. 47 mm and a pore size of 0.22 µm from Merck Millipore Ltd. (Darmstadt, Germany).

### 2.2. Procedures 

#### 2.2.1. CNF Production

The cellulose nanofibers (CNF) suspension 1.328 wt% was obtained by green and environmental process using specific enzymes, to avoid the use of toxic and expensive materials [16]. 

Hence, CRM was initially mechanically beaten at 1000 revolutions in a PFI-mill to increase the swelling in water and make the cellulose accessible for the enzyme. Then an enzymatic pretreatment was carried out using 3% (*w*/*w*) of CRM dispersed in 50 mM tris (hydroxymethyl)aminomethane/HCl buffer with pH 7 and, 3% (*w*/*w*) of enzyme load (Novozym 476, endoglucanase by Novozymes A/S, (Bagsværd, Denmark) at 50 °C for 2 h. Once the reaction was finished, the enzyme was denatured and incubated again at 80 °C for 30 min. Finally, the CNF result was washed again with distilled water and beaten at 4000 revolutions in a PFI-mill.

#### 2.2.2. Pure CNF Films

Pure CNF films of 0.04 g dry weight were produced (Figure S1a). Hence, 3.012 g of CNF suspension (1.55 wt%) was diluted with 50 mL of distilled water, and the solution obtained was sonicated using a Branson Sonifer 450 equipment (Emerson, Barcelona, Spain) for 30 s, with breaks of 10–20 s repeatedly until the dispersion was homogeneous. Subsequently, the mixture obtained was vacuum filtered using a filtration assembly for 47 mm filters with glass support and a nylon membrane with a pore size of 0.22 µm. The filtration took approximately 1 h and 15 min. Therefore, the wet cake generated was removed and allowed to dry at room temperature (~25 °C) [17].

#### 2.2.3. Preparation of CNF/rGO Nanocomposite Films

The procedure for the elaboration of CNF/rGO was carried out using different loading of GO: 0.5%, 1%, 2.5%, 5%, 10%, 15%, 50%, and 100% (Figures S1b, S3, and Table S1). Likewise, to improve the conductive properties of nanocomposites the rGO reaction was performed once the GO has been introduced into the matrix/support of the film.

Therefore, GO dispersion was performed by adding 30 mL of distilled water and stirring for 5–10 min. Then the CNF suspension (1.55 wt%) and GO dispersion were mechanically mixed and sonicated repeatedly until a homogeneous suspension was obtained. Besides, the suspension result (CNF/GO) was vacuum filtrated with nylon membranes (size pore 0.22 µm) in a period time of 2–24 h depending on the loading of the CNF/GO, and then all the samples were dry for 24 h at room temperature (~25 °C). 

Finally, the reduction of GO to rGO was carried out by placing each of the obtained dried CNF/GO films in a glass petri dish and adding 57 wt% hydroiodic acid. The reaction was performed at room temperature (~25 °C) for 1 h according to the literature [18] with a minor modification. The CNF/rGO films were rinsed with deionized water to remove all excess acid and finally, all the films were dried at room temperature (~25 °C) for 24 h to remove the moisture and stored until the analysis (Figure S1b). 

#### 2.2.4. Preparation of CNF/PEDOT:PSS Nanocomposite Films

The CNF/PEDOT:PSS samples were prepared using different loadings of PEDOT:PSS: 1%, 5%, 10%, 15%, 20%, 30%, 40%, and 50% (Figures S1c, S4, and Table S2). Firstly, once CNF suspension (1.55 wt%) was ready (as described in Section 2.2.1), the necessary amount of the PEDOT:PSS suspension was weighed (Table S2) and diluted with 30 mL of distilled water, then the resulting dispersion was kept stirring for 5–10 min. Then, both dispersions (CNF and PEDOT:PSS) were mixed and sonicated for 30 min, until a homogeneous dispersion was obtained. he mixture was vacuum filtrated with nylon membranes (size pore 0.22 µm) in a period time of ~1.30 h to 3 h subject on the CNF/PEDOT:PSS composition and then dried for 24 h at room temperature (~25 °C) until their use (Figure S1c). 

#### 2.2.5. Preparation of CNF/PEDOT:PSS/rGO Nanocomposite Film

The ternary nanocomposites were performed according to the composition described in Table 1. Hence, CNF, GO and PEDOT:PSS suspensions were done separately and sonicated for 30 min until a homogeneous mixture was obtained. Later, the suspensions obtained were placed in the glass beaker and sonicated for 30 s until the dispersion became homogeneous. Then, CNF/GO/PEDOT:PSS dispersion was filtered (using 47 mm diameter nylon membrane with pores 0.22 µm) for around 2–8 h according to the CNF/PEDOT:PSS/rGO composition. Subsequently, the film filtered was placed in a glass Petri dish and a few drops of hydroiodic acid 57 wt% were added covering the entire film from top to bottom. It is left in this way for 1 h at room temperature (~25 °C) for the reduction of GO to rGO to take a place. Finally, the film CNF/PEDOT:PSS/rGO was washed several times with distilled water to eliminate all the excess acid and dry at room temperature for 24 h (see Figure S2). 

**Figure 1:**
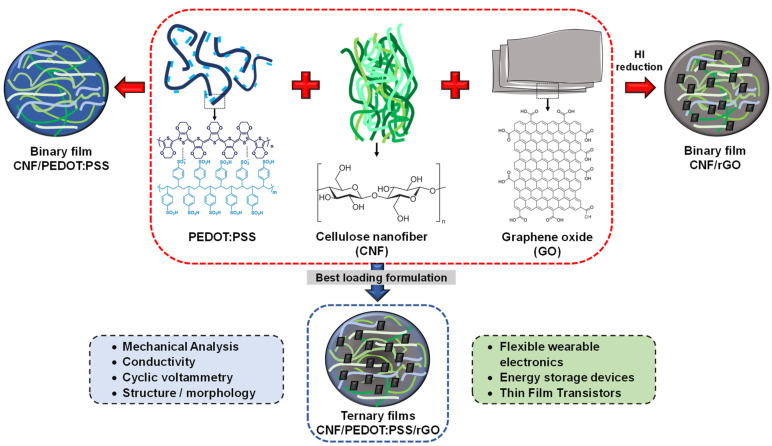
General scheme of the production process of binary and ternary films.

A general scheme to produce the binary and ternary formulations is presented in Figure 1.

### 2.3. Characterization 

#### 2.3.1. Mechanical Properties 

The mechanical properties of the film’s samples with various thicknesses and a rectangular size of 34 × 5 mm were measured using a Mechanical tester Instron 5565 A equipment (Instron, Norwood, MA, USA), with a crosshead speed of 6 mm/min. The relative moisture of 50% and room temperature (~25 °C) were kept during the measurement [19]. In addition, at least five different tests are carried out for each type of sample, to obtain the statistical error in accordance with the ISO 527-1:2019 standard [20]. 

#### 2.3.2. Sheet Resistance and Conductivity Test

To determine the conductivity of the film obtained, the samples were cut into a rectangular shape of 5 × 17 mm, and the electrical conductivity was measured using a Keithley 2450 Sourcemeter (Tektronix, Oldbury, UK) connected to an Everbeing’s Four-Point-Probe SR-4 (Micro Point Pro, Yokneam, Israel). The sheet resistance of the samples (*Rs*) was attended by Equation (1) [15]. For each experiment, ten different tests were carried out.

(1) $$Rs=\frac{\pi }{Ln2}\cdot R_{measured}\cdot C$$

where *C*: correction factor = 0.61035 (Table S3).

Further, the conductivity ρ can be calculated from Equation (2), as follows:

(2) $$\rho =\frac{1}{R_{s}\cdot t}$$

where t is the thickness of the sample.

#### 2.3.3. Cyclic Voltammetry 

The cyclic voltammetry (CV) with a standard three-electrode electrochemical cell using a Potentistat BioLogic SP-300 equipment (BioLogic, Seyssinet-Pariset, France) was used. All the experiments were carried out employing the fabricated film samples of 5 × 17 mm as a working electrode in a 2M NaCl-saturated electrolyte Ag/Ag Cl as reference electrode and a platinum wire counter electrode. The cyclic voltammograms were recorded in the potential window of −0.9 to +0.9 V vs. Ag/AgCl at different scan rates: 5, 20, 50, 100, and 200 mV/s. As well as capacitance is the ability of a component to collect and store energy in the form of an electrical charge. This work analyzed the specific capacitance, *C_sp_* following the equation (Equation (3)).

(3) $$C_{sp}\left(F\cdot g^{-1}\right)=\frac{i}{m\cdot v\cdot \Delta V}$$

where i = is the integration in the CV curve; v  = the scan rate in $V\cdot s^{-1}$; m  = the mass in grams of the electrode material and ΔV = 1.8 V (potential window).

#### 2.3.4. SEM Analysis and Raman Spectroscopy

The morphology of the films was analyzed by a scanning electron microscope (JEOL JSM-7800F Prime, Tokyo, Japan). The instrument has a field emission gun and is also equipped with an EDX (energy dispersive X-ray) detector for chemical analysis, a STEAM (scanning transmission electron microscopy) detector for thin foil analysis, and a SXES (soft X-ray emission spectrometer) for analysis of low energy X-rays (around 50–200 eV).

The Raman spectroscopy was analyzed using a Raman spectrometer (Horiba Jobin Yvon LabRAM, Kyoto, Japan) directly on the films without requiring any special preparation and that does not entail any alteration of the surface on which the analysis is carried out.

## 3. Results and Discussion

### 3.1. Binary Nanocomposite Films

The binary films were prepared and successfully processed via a simple, scalable, and low-cost method through vacuum filtration followed by reduction of GO only in the case of CNF/rGO films (Figure 1). Subsequently, Figures S3 and S4 show the film production in binary form using rGO and the conductive polymer PEDOT:PSS, respectively. Likewise, Figure S3a demonstrated the achievement in homogeneous CNF/GO films, this might be due to CNF having a strong attractive interaction with GO via hydrogen bonding and van Waals interaction [18].

**Figure 2:**
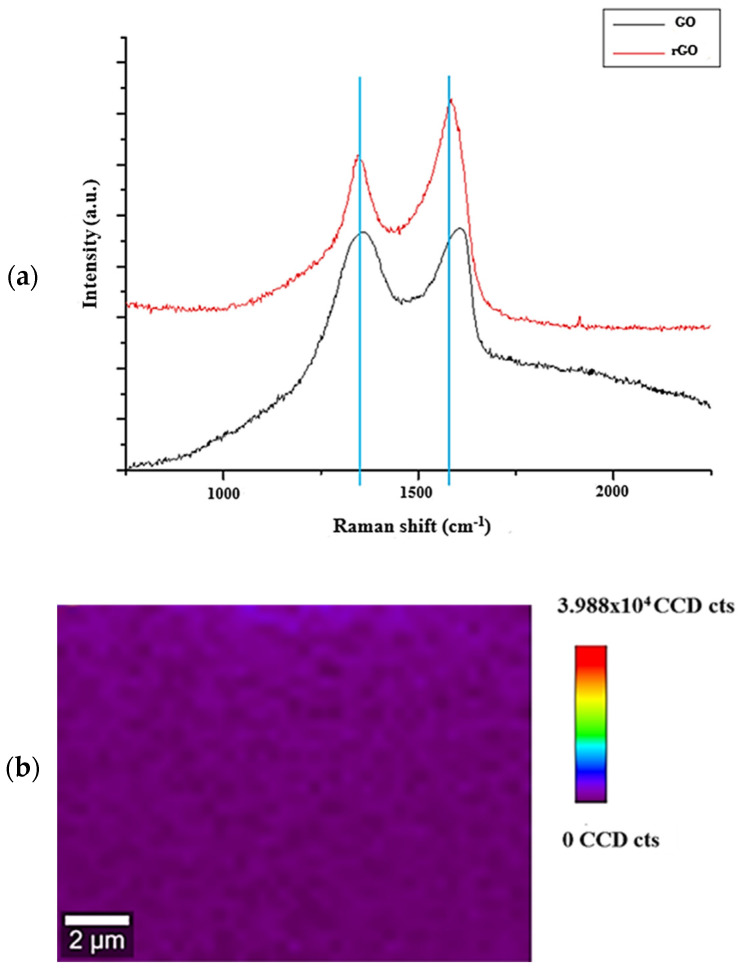
(**a**) Raman spectra of GO and rGO and (**b**) Raman image of CNF/rGO 15% nanocomposite, CCD for charged-coupled device detector.

In addition, Figure S3b shows the CNF/rGO films obtained through the reduction reaction of GO to rGO where visually a color change occurs since the films become darker. Figure 2 shows the Raman spectra in the spectral range of 1000 cm^−1^ to 1800 cm^−1^ which confirms the reduction reaction. The comparative spectroscopic analysis of GO and rGO reveals significant modifications in Raman peak intensities due to the acid treatment that resulted folding of structures, impurities, hybridization structures (sp^1^, sp^2^, and sp^3^), lattice contraction, and exfoliation [21]. The changes in the carbon materials by the analysis of prominent peaks at 1360 cm^−1^ (caused by the graphite edges) are related to the breathing modes of sp^2^ atoms in rings and this phenomenon is activated by the presence of defects or disorder in the graphene structure. In this sense, the intensity of the D band increases with the level of disorder, which is why it is more prominent in GO compared to rGO_2_ [22]. 

The peak at 1560 cm^−1^ (in-phase vibration of the graphite lattice), corresponds to the characteristic band G [23]. It is associated with the E_2_g phonon at the Brillouin zone center, indicating the presence of graphitic domains. Therefore, an increase in the band for rGo (red line) demonstrated an increase in sp^2^ cluster size and more structural defects during the reduction process [18,22].

**Figure 3:**
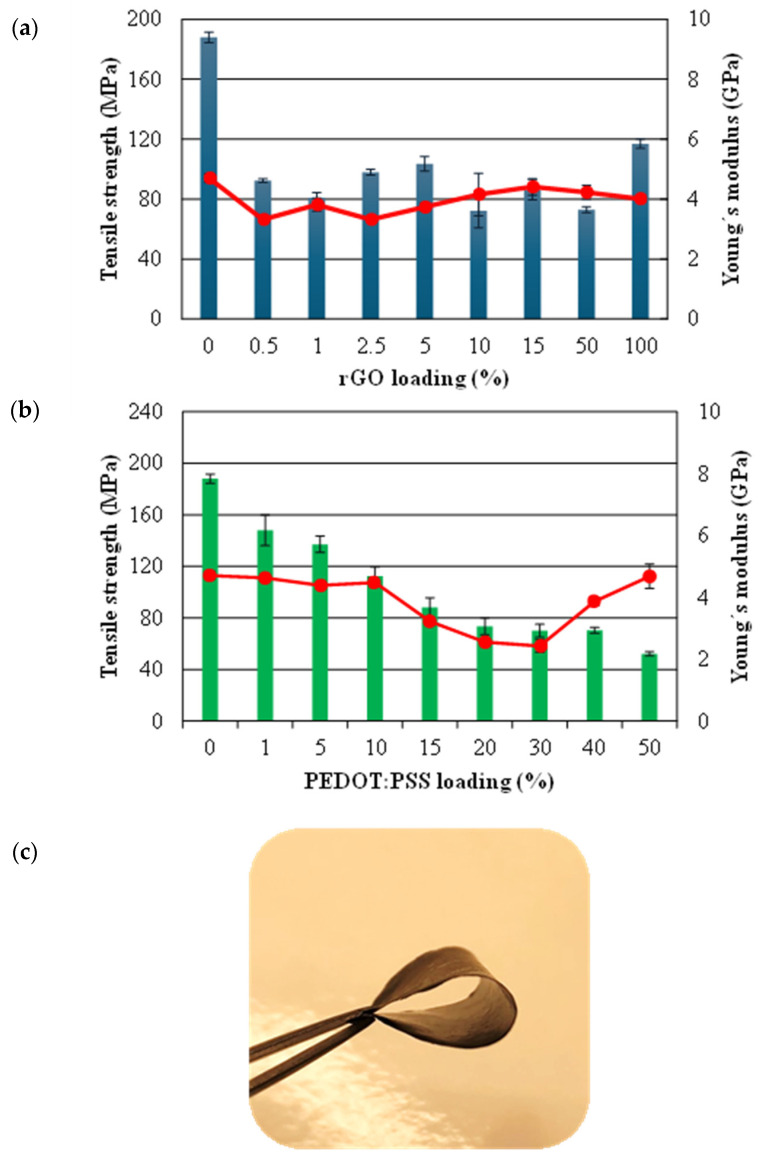
Mechanical properties of (**a**) CNF/rGO films (0.5–100% wt) and (**b**) CNF/PEDOT:PSS films (1–50% wt). (**c**) Photograph of the CNF/rGO film showing the flexibility. In the graph, the bars represent the tensile strength and the solid line the Young’s modulus.

The results showed (Figure 3a,b) an outstanding tensile strength of 187.99 ± 3.60 MPa and Young’s modulus of 4.71 ± 0.14 GPa in CNF pure, as compared to bibliography for different films [15,24]. These properties can change depending on the cellulose source and preparation method employed. 

In addition, it was observed that the mechanical strength of the films with binary formulation decreased with increasing rGO and PEDOT:PSS loadings several orders of magnitude (see Figure 3a,b). Consequently, the tensile strength was reduced to 92.44 ± 1.25 MPa for 0.5% wt rGO, which could be related to the weakening of the hydrogen bonding in the rGO after chemical reduction using HI [19]. In contrast, 1% wt PEDOT:PSS addition only reduced approx. 20% of the tensile strength (147.95 ± 11.92 MPa).

On the other hand, Figure 3a shows that there was not a dramatical reduction of Young’s modulus for CNF/rGo. The values for 5% and 15% wt films were 3.74 ± 0.13 and 4.41 ± 0.27 GPa, respectively. The slight decrease in the strength can be correlated with the reduced elongation of the composite paper CNF/rGo as compared with the pure cellulose paper [23].

Figure 3b also demonstrated a constant value of elastic modulus for CNF/PEDOT:PSS films for 5%, 10%, and 40% wt. However, a significant difference could be observed for CNF/PEDOT:PSS 50% wt films with respect to pure CNF, which could be linked to the fact that PEDOT:PSS exhibits good flexibility and stretchable properties [25]. Moreover, the current work improved results s compared to other authors [15], which used CNF/PEDOT:PSS (53.5% wt) post-treated with DMSO and had a Young´s modulus of 1.87 GPa. Therefore, it ca be highlighted that this study can emphasize the development of processing strategies for the formation of mechanical robust and flexible film.

The flexibility of the produced films can be observed from Figure 3c. The general improvement in mechanical properties can be attributed to the sonification process, deleting all possible air in nanofibrils suspension, and to the filtration, promoting some nanofibrils alignment resulting in a homogeneous structure with low porosity and high mechanical properties [24]. Also, the good dispersion of graphene in nanocomposite films due to the optimum loading could have generated a synergistically improved strength [26].

**Figure 4:**
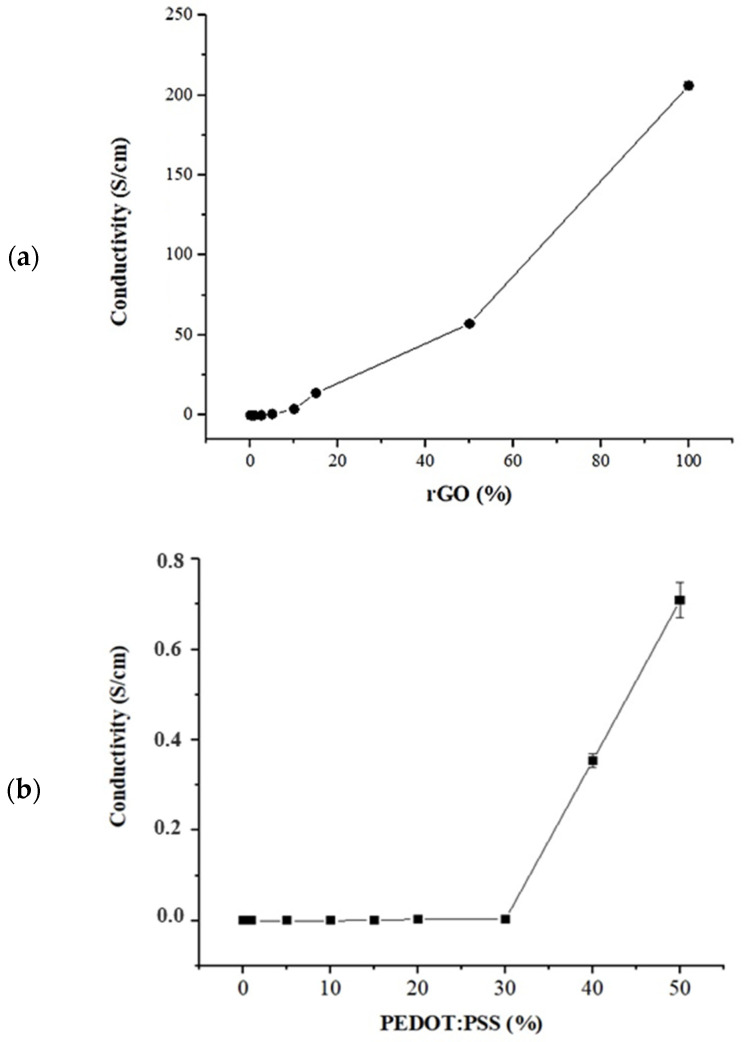
Characterization by the conductivity of (**a**) CNF/rGO under different loadings (0–100%) and (**b**) CNF/PEDOT:PSS under different loadings (0–50%).

Whereas pure CNF is not electrically conductive, CNF/rGO and CNF/PEDOT:PSS exhibited conductivity. Figure 4 shows that the conductivity increased progressively with the augment of rGO and PEDOT:PSS in the formulation of the films. The conductivity of CNF/rGO film with 0.5% wt was 3.14 × 10^−9^ ± 2.78 × 10^−10^ S/cm and for the PEDOT:PSS 1% wt was 1.4 × 10^−9^ ± 8.4 × 10^−11^ S/cm. Instead, for rGO loading is 50% wt the conductivity was 57.20 ± 0.75 S/cm; attributed to the good connection between graphene sheets and the success of the reduction of GO to rGO. Employing hydroiodic acid meant that the sp^2^ structure of GO was partly restored, and the conductivity of the film improved with the increase of rGO load [19]. In addition, the 100% rGO sample achieved values of ~185 S/cm. These results showed improvement with respect to other authors [18,27].

The current study can contribute to obtaining a sensor with enough conductivity properties using sustainable and simple processes. Other recent studies have demonstrated the application of polymer electrolytes with excellent anodic and mechanical properties such as (PVsF(HFP)) [28] and the application of ionic liquid and TEMPO-oxidized as cellulose pretreatment [29] in improving gel electrolyte layer and gel actuators, respectively. However, this process involves the use of expensive and toxic reagents which avoid the eco-friendly and scalable process. Different researchers reported around 8.5 ± 0.5 and 7.2 ± 0.2 S/cm [29] and 17.5 S/cm [28].

However, in the present study, high conductivity was already found CNF/PEDOT:PSS 50% wt (7.1 × 10^−1^ ± 3.9 × 10^−2^ S/cm). The difference in conductivity of rGO and PEDOT:PSS can also be due to the PSS group which confers insulating properties, and which can reduce the conductivity of CNF/PEDOT:PSS samples [30].

In addition, as shown the Figure 3, the CNF/rGO 50% wt and CNF/PEDOT:PSS 50% wt showed lower mechanical strength properties (tensile strength and Young´s modulus) that could limit its practical application. Remarkably, the CNF/rGO 5% wt, 15% wt, and CNF/PEDOT 40% wt still exhibited relatively superior conductivity of 0.76, 13.88, and 0.35 S/cm, respectively, as well as relatively superior mechanical properties. Consequently, CNF/rGO 15% wt with 13.88 S/cm was higher than most of the results of conducting polymer and CNF-based film reported in the literature (for instance, the results of bacterial cellulose/GO 1.1 × 10^−6^ S/cm, rGO/CNF 0.718 S/cm [31], CNF/PEDOT:PSS 2.58 S/cm [24] and CNF/Polypyrrole 4.3 S/cm) [32].

Therefore, considering the mechanical strength and conductivity achieved in the experiments using CNF/rGO 5% and 15% wt, which were more suitable as practical applications for devices engineering applications, were selected to evaluate ternary films. Moreover, as the results did not demonstrate a significative high conductivity using PEDOT:PSS, the effect between the two conductive materials will be analyzed to improve this property in ternary films. Hence, the percentages of 15, 20, and 40 wt of PEDOT:PSS were considered to be functional since these formulations showed adequate values of conductivity and mechanical properties.

**Figure 5:**
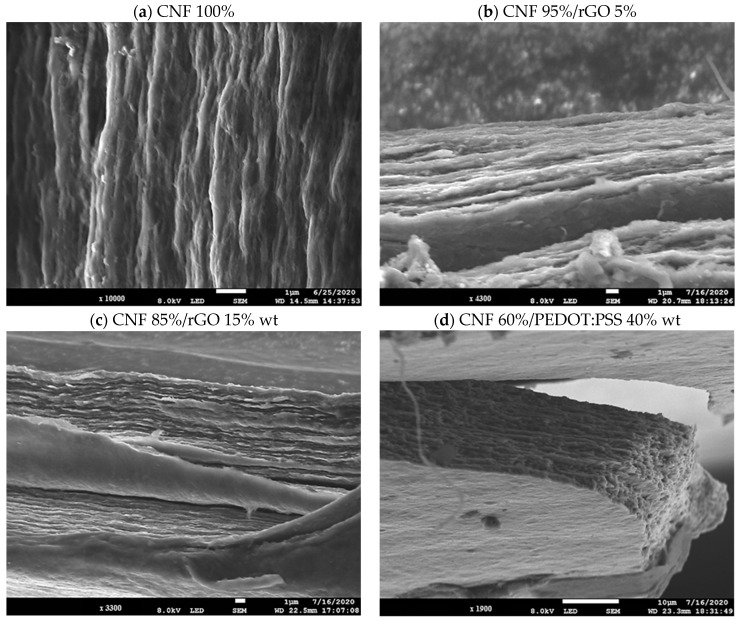
Cross Section SEM images taken with variable magnification: (**a**) ×10,000; (**b**) ×4300; (**c**) ×3300 and (**d**) ×1900. All the percentages are present in % wt.

On the other hand, the morphological properties of CNF/rGO and CNF/PEDOT:PSS were examined by Cross-section SEM. Hence, Figure 5a shows the cross-section SEM image of pure CNF film, demonstrating a compact multilayer configuration of interconnected cellulose nanofibers, in consequence this could contribute to the high mechanical performance of pure CNF film due to the strong interactions between nanofibrils, and nanofibrils entanglements [33].

Instead, the CNF/rGO 5% and 15% wt film (Figure 5b,c) shows a CNF that is well adhered and interconnected with the rGO flakes. This proves detached fragments or cracks, as noticed by SEM. In addition, it was indicated that rGO was successfully coated in CNF substrate, due to CNF/rGO film exhibiting a relatively rough and wrinkle-like structure, which could be attributed to the hydrophobic property of rGO [18].

In contrast, Figure 5d, which corresponds to CNF/PEDOT:PSS 40% wt film, demonstrates a robust and even more compact multilayer configuration of interconnected cellulose nanofibers. It has less space between CNF than previously demonstrated when rGO was used. This could be due to PEDOT:PSS, which can be successfully assembled under a directional flow by vacuum filtrating [31].

### 3.2. Ternary Nanocomposite Films

**Figure 6:**
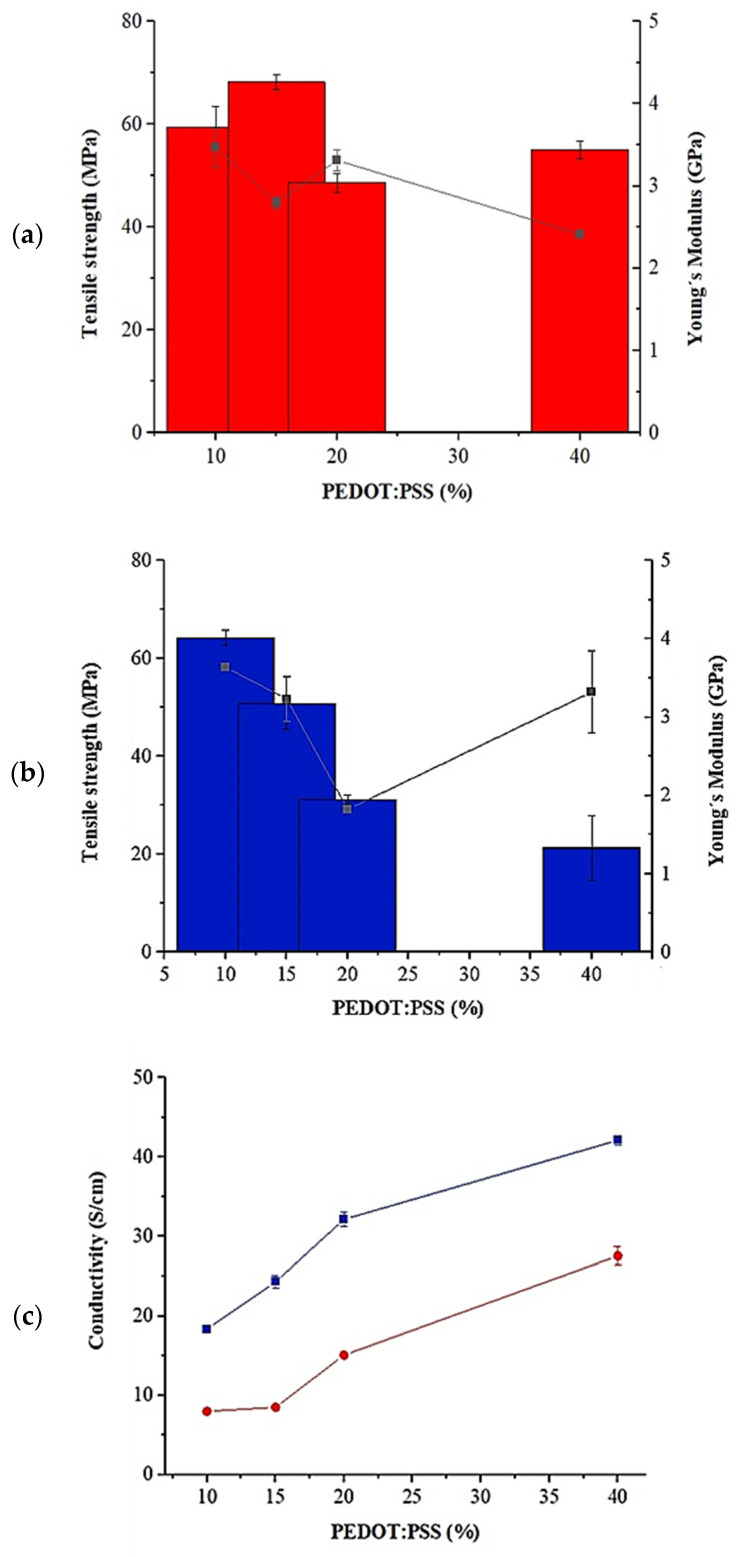
Characterization of ternary films composted by CNF/rGO/PEDOT:PSS by Tensile strength and Young´s modulus (**a**) using 5% rGO and (**b**) 15% rGO and PEDOT:PSS between 10–40%. (**c**) Conductivity properties using 5% (red line) and 15% (blue line) of rGO and PEDOT:PSS between 10–40%. In the graph, the bars represent the tensile strength and the solid line Young’s modulus.

Figure 6a shows the tensile strength and Young´s modulus obtained for CNF/rGO/PEDOT:PSS formulations. The maximum value of tensile stress was (68.24 MPa) and an elastic modulus of 2.80 ± 0.08 GPa was achieved using CNF80%/rGO 5%/PEDOT:PSS 15% wt (see Figure 6a). The strength and flexibility property was slightly reduced compared with the binary films. However, this could be attributed to the hydrogen bonding among CNF connected with residual oxygen-containing groups of rGO sheets and the hydrogen bonds formed between CNF and PEDOT chains, which could interact with the carboxylic group (COO^−^) of CNF. Moreover, the negatively charged chains of PSS (SO_3_^−^), which may interpose between cellulose nanofibrils, consequently limit the number of CNF inter-fibril-bond OH interactions and generate a significant decrease in the mechanical properties [24,34,35].

Figure 6b shows the conductivity property obtained by the combination of CNF/rGO/PEDOT:PSS in which a substantial improvement of this property is clearly visible over binary formulation. The maximum value achieved used CNF/rGO/PEDOT:PSS with a load of 45/15/40 (% wt) (42.22 S/cm); this is an improvement of 120.32, which was 55.40 y 3.03 higher than that obtained by CNF/PEDOT:PSS 40% wt, CNF/rGO 5% and 15%, respectively. This enhancement can be justified by the fact that hydroiodic acid could dissolve part of the additional PSS shell (insulating) to release the conductive PEDOT [36]. The electrical properties of PEDOT:PSS can vary according to the doping process [37]. Also, it has been discovered that different post-processing methods (DMSO, co-solvents, acid agent, and ionic liquid) lead to an improvement in the electrical conductivity by reducing the Coulombian interaction between the charge carriers in the PEDOT chains and the PSS negative ions, which generates a face separation or induces a morphological reorganization [30]. In addition, the enhancement in the electrical conductivity confirms that rGO is homogeneously dispersed to form a continuous network in the nanocomposite [31].

In addition, this result is similar to does report by Kuzmenko et al. [38] that obtained 49 S/cm in CNF/rGO films. Likewise, Hou et al. [18] inform the conductive results of 43.82 S/cm using CNF/GO film paper with sandwich structure. In addition, better results were obtained than the report by Yang et al. [39] achieved nearly 41 S/cm, and Lay et al. [24] for CNF/PEDOT:PSS/Polypyrrole with a conductivity of 10.55 S/cm.

**Figure 7:**
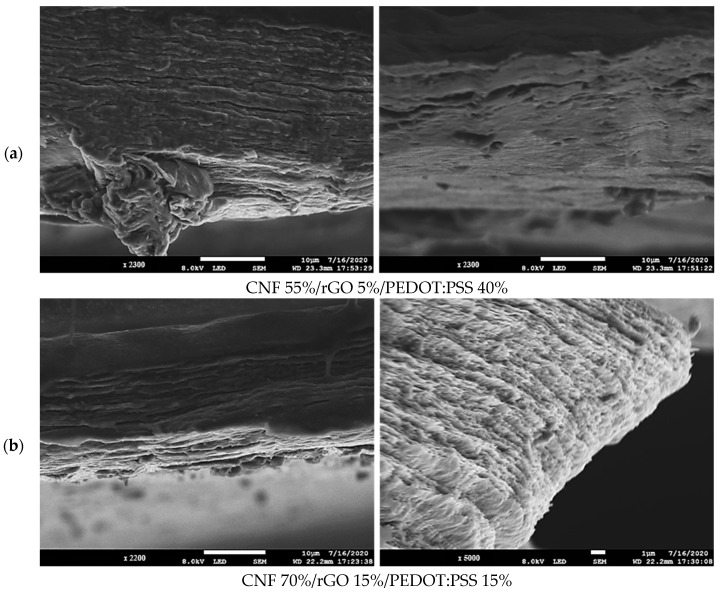
SEM cross-section images of ternary films.

The morphology of the ternary films’ formulations obtained was analyzed by SEM. As shown in Figure 7a,b, and its compact structure, it is possible to appreciate that this could be related to the success of the sonication step, due to the removal of the air in the nanofibrils’ suspension. In addition to the filtration process, which might have helped the nanofibrils’ alignment [24]. Moreover, it is observed that both nanocomposite films possess a porous structure which could improve the electrical conductivity [39]. Also, it could detect a smoother surface and a denser interlayer structure in Figure 7a than in Figure 7b, which is probably because the holes between the CNFs were filled with more load of PEDOT:PSS (40% wt). In contrast, Figure 7b presents a slightly rough structure, which could be related to a higher amount of rGO.

**Figure 8:**
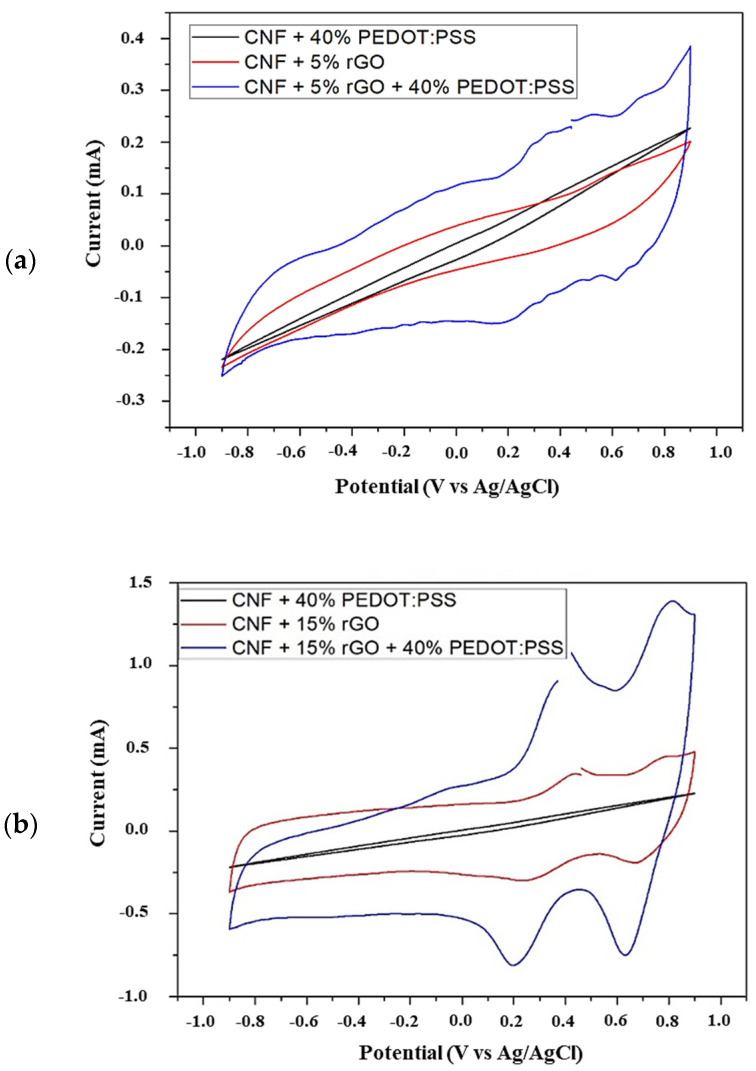
Electrochemical analysis by CV curves of binary and ternary films formed by CNF/rGO/PEDOT:PSS for (**a**) 5% and (**b**) 15% of rGO, as energy-storage electrode materials in 2 M NaCl solution at a scan rate of 5 mV·s^−1^.

Cyclic voltammetry (CV) tests have been achieved to examine the effect on specific capacitances, the electrochemical behavior as well as, the potential application as transparent conductive electrodes for energy storage applications. Hence, the measurements were conducted in a three-electrode configuration using 2 M NaCl as the electrolyte. In this sense, Figure 8a illustrates CV curves of CNF55%/rGO 5%/PEDOT:PSS 40% wt and Figure 8b CNF 45%/rGO 15%/PEDOT:PSS 40% wt, formulation, respectively. 

Therefore, Figure 8 shows the Faradaic nature of the charge storage mechanism in these samples, with one (Figure 8a) and two (Figure 8b) double redox peaks, probably associated with the remaining oxygen functional groups existing on the rGO layers [34].

Besides, the film constituted by CNF 60%/PEDOT:PSS 40% wt (black line) presents a significant resistance due to the low conductivity of the film. However, redox peaks can be clearly recognized in the CNF 85%/rGO 15% wt binary formulation and CNF/rGO /PEDOT:PSS 55%/5%/40% and 45%/15%/40, respectively, in ternary films. 

On the other hand, the peak current and area under the curve also increases when the amount of rGO increases from 5% to 15%, keeping the amount of PEDOT:PSS constant at 40% (Figure 8a,b and Figure S5). This could be related to the absence of a binder material and abundant conductive interconnections between rGO sheets and CNFs might facilitate the effective charge transfer throughout the film. Also, it may be related to the reorganization of rGO sheets during the intercalation of electrolyte ions, which leads to a higher accessible surface area increasing the electrochemical behavior [37].

Moreover, Figure 8a,b illustrate the peak current intensities with remarkably increased in the ternary nanocomposite formulation (blue line) which can be correlated to enhanced electrical conductivity and possible synergistic effects between the rGO and PEDOT:PSS in ternary films. This is because CNF could be attached to a 2D rGO and a1D PEDOT:PSS building, and a 3D structure of interconnected pores, which could improve the ionic transport pathways [18,24].

In addition, the charge stored during forward and backward scans directly corresponds to the area under the CV curves (see Equation (3)), therefore, the highest capacitance was obtained for the CNF 45%/rGO 15%/PEDOT:PSS 40% wt film at 5 mV·s^−1^, as shown in Table 2. Moreover, it can be observed that the capacitances decrease accordingly with increasing the scan rate due to insufficient time for the electrolyte ions to complete the electrochemical reaction at higher scan rates. Hence, it could highlight the ability of energy storage and electrochemical performance of the films CNF55%/rGO5%/PEDOT:PSS40% wt obtained in this study.

The charge stored during forward and backward scans directly corresponds to the area under the CV curves (see Equation (3)), therefore, the highest capacitance was obtained for the CNF 45%/rGO 15%/PEDOT:PSS 40% wt film at 5 mV·s^−1^, as shown in Table 2. Moreover, it can be observed that the capacitances decrease accordingly with increasing the scan rate. This is due to the insufficient time for the electrolyte ions to complete the electrochemical reaction at higher scan rates. Hence, it could be noted that the peak current and area under the curve also increase when the amount of rGO increases from 5% to 15%, keeping the amount of PEDOT:PSS constant at 40% (Figure S5). 

Hence, it could highlight the ability of energy storage and electrochemical performance of the films CNF/rGO/PEDOT:PSS (55/5/40 and 45/15/40% wt) obtained in this study.

In this sense, according to the results obtained in this work, the application of this formulation in flexible and portable electronics. In addition, its application in supercapacitors, batteries, and flexible solar cells should be highlighted, due to its high power, good cyclability and flexibility, and mechanical properties achieved. This study also presents a different material that can contribute to sustainability efforts, such as reducing carbon footprints or being recyclable due to the origin of its composition. 

## 4. Conclusions

This study presents an efficient process to obtain flexible and conductive nanocomposites using sustainable methods for the development of green electronic devices. In particular, binary and ternary nanocomposite formulations from cellulose nanofibers (CNF), reduced graphene oxide (rGO), and poly(3,4-ethylenedioxythiophene)-poly(styrenesulfonate) (PEDOT:PSS) conductive polymer were produced using a simple and scalable manufacturing process. SEM microphotographs and Raman spectroscopy demonstrated a homogeneous distribution of the conductive elements in the CNF matrix, which contributed to the improvement in electrical and electrochemical properties. Also, a synergy effect was observed in the ternary formulations blend between rGO and PEDOT:PSS in CNF. The reduction of graphene oxide (GO) using hydroiodic acid enhanced the ion diffusion, which thus contributed to the improvement of conductivity in the ternary nanocomposites’ films. As a result, the present study provides insights for developing bio-based stretchy and conductive electronic devices using sustainable and eco-friendly alternatives.

## Figures and Tables

**Table 1:** Reactant composition in the formulation of ternary films.

CNF (%)	rGO (%)	PEDOT:PSS (%)	CNF Suspension (g)	GO Suspension (g)	PEDOT:PSS Solution (g)
85	5	10	2.56	0.50	0.12
80	5	15	2.41	0.50	0.17
75	5	20	2.26	0.50	0.23
55	5	40	1.66	0.50	0.46
75	15	10	2.26	1.50	0.11
70	15	15	2.11	1.50	0.17
65	15	20	1.96	1.50	0.23
45	15	40	1.36	1.50	0.46

The theoretical dry weight of the films was 0.040 g in all the experiments; CNF (1.328 wt%); GO (0.4 wt%); PEDOT:PSS (3–4 wt%).

**Table 2:** Capacitances of CNF/rGO/PEDOT:PSS with different loads (wt %).

Loading (%)			Capacitances (F/g)				
rGO	PEDOT:PSS	CNF	5 m V/s	20 m V/s	50 m V/s	100 m V/s	200 m V/s
5	-	95	18.32	7.20	3.56	1.91	1.01
-	40	60	22.00	5.90	2.45	1.26	0.68
5	40	55	28.12	17.35	13.28	10.76	8.21
15	-	85	44.68	33.55	15.98	20.70	13.90
-	10	90	18.38	8.90	5.06	3.20	1.96
15	10	75	84.07	68.87	47.16	36.97	27.68
15	-	85	44.68	33.55	15.98	20.70	13.90
-	40	60	22.00	5.90	2.45	1.25	0.68
15	40	45	100.19	60.08	43.88	33.16	23.40

## Data Availability

The raw data supporting the conclusions of this article will be made available by the authors on request.

## Supplementary Materials

The following supporting information can be downloaded at https://www.mdpi.com/article/10.3390/polym16213035/s1: Figure S1: Laboratory procedure for (a) pure nanocomposite production, (b) CNF/rGO nanocomposite; (c) CNF/PEDOT:PSS nanocomposite; Figure S2: Laboratory procedure for CNF/PEDOT:PSS/rGO ternary nanocomposites; Figure S3: Pictures of the CNF/rGO nanocomposites (a) before and (b) after the reduction reaction; Figure S4: Pictures of the CNF/PEDOT:PSS nanocomposites; Figure S5: Electrochemical analysis by CV curves of ternary nanocompostes formed by CNF/rGO/PEDOT:PSS under different load (% wt) as energy-storage electrode materials in 2 M NaCl solution at different scan rates; Table S1: CNF and rGO nanopapers composition; Table S2: CNF and PEDOT:PSS nanopapers composition; Table S3: Geometric correction factors.
