# Supplementary material for: Advanced Flexible Wearable Electronics from Hybrid Nanocomposites Based on Cellulose Nanofibers, PEDOT:PSS and Reduced Graphene Oxide
Source: Polymers (Basel). 2024 Oct 29;16(21):3035. doi: 10.3390/polym16213035 (PMC11548421; doi:10.3390/polym16213035)
Supplement: Supplementary file 1 [file polymers-16-03035-s001.zip › polymers-3077869-supplementary.pdf]

Supplementary data

Advanced Flexible Wearable Electronics from Hybrid Nanocomposites Based on Cellulose Nanofibers, PEDOT:PSS and Reduced Graphene Oxide

Ana Carrascosa [1], Jaime S. Sánchez [2,3], María Guadalupe Morán-Aguilar [4], Gemma Gabriel [5,6] and Fabiola Vilaseca [1,4,7,*]

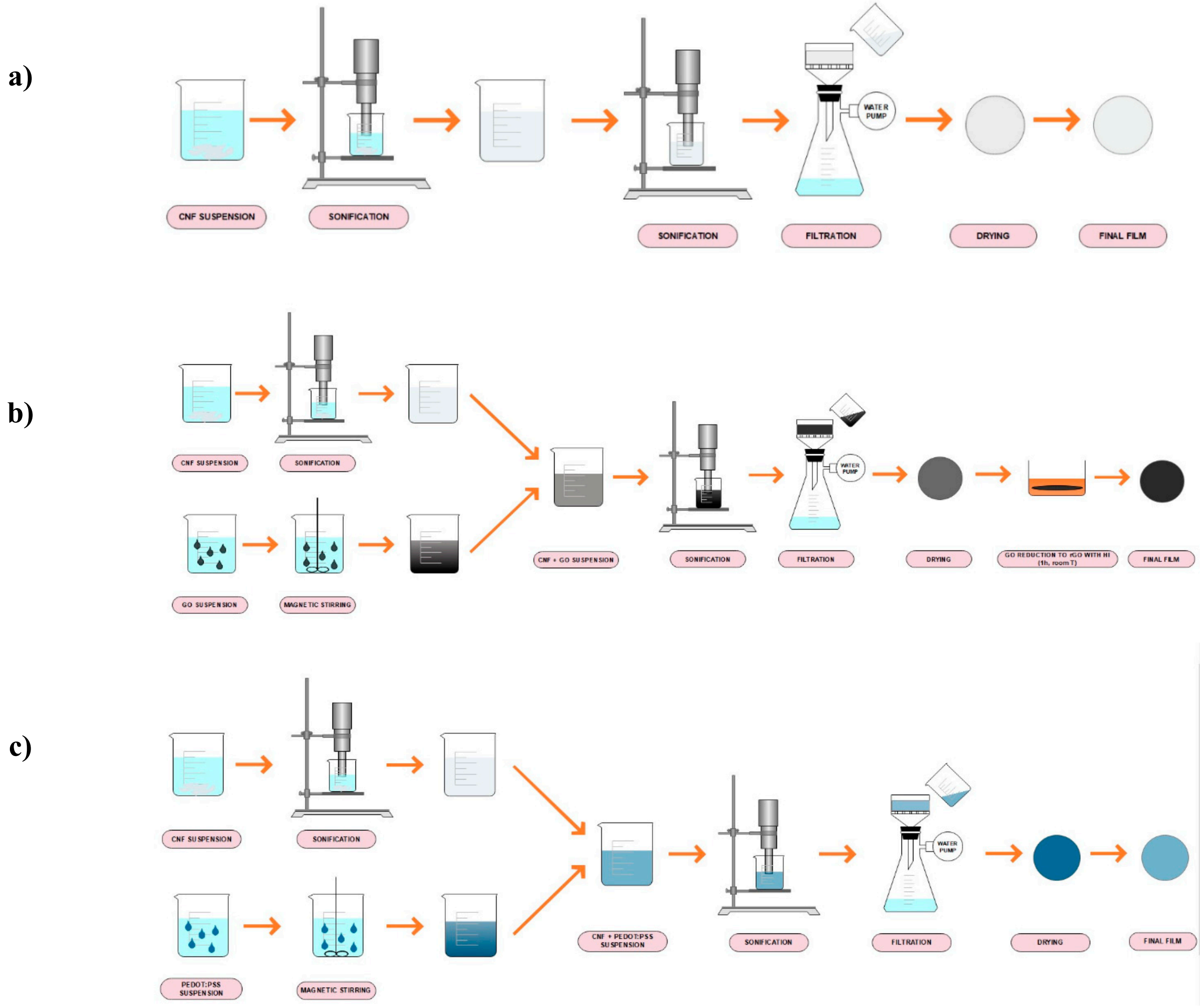

Figure S1. Laboratory procedure for a) pure nanocomposite production, b) CNF/rGO nanocomposite; c) CNF/PEDOT:PSS nanocomposite.

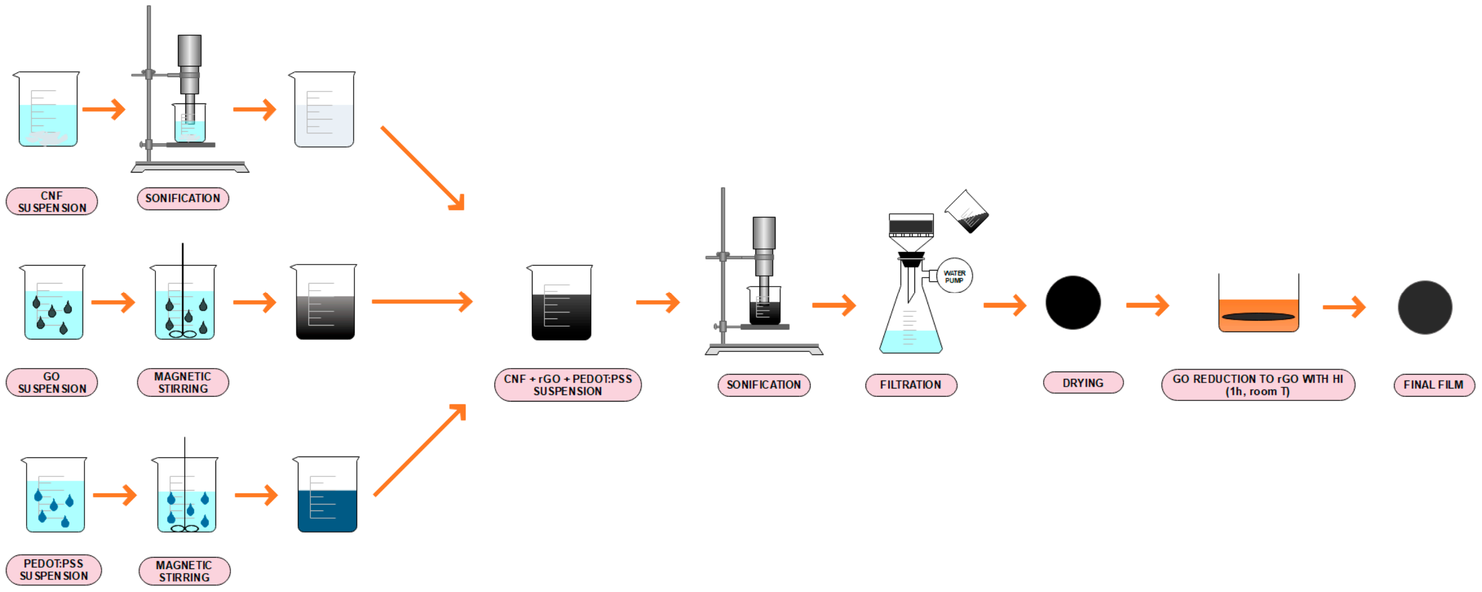

Figure S2. Laboratory procedure for CNF/PEDOT:PSS/rGO ternary nanocomposites.

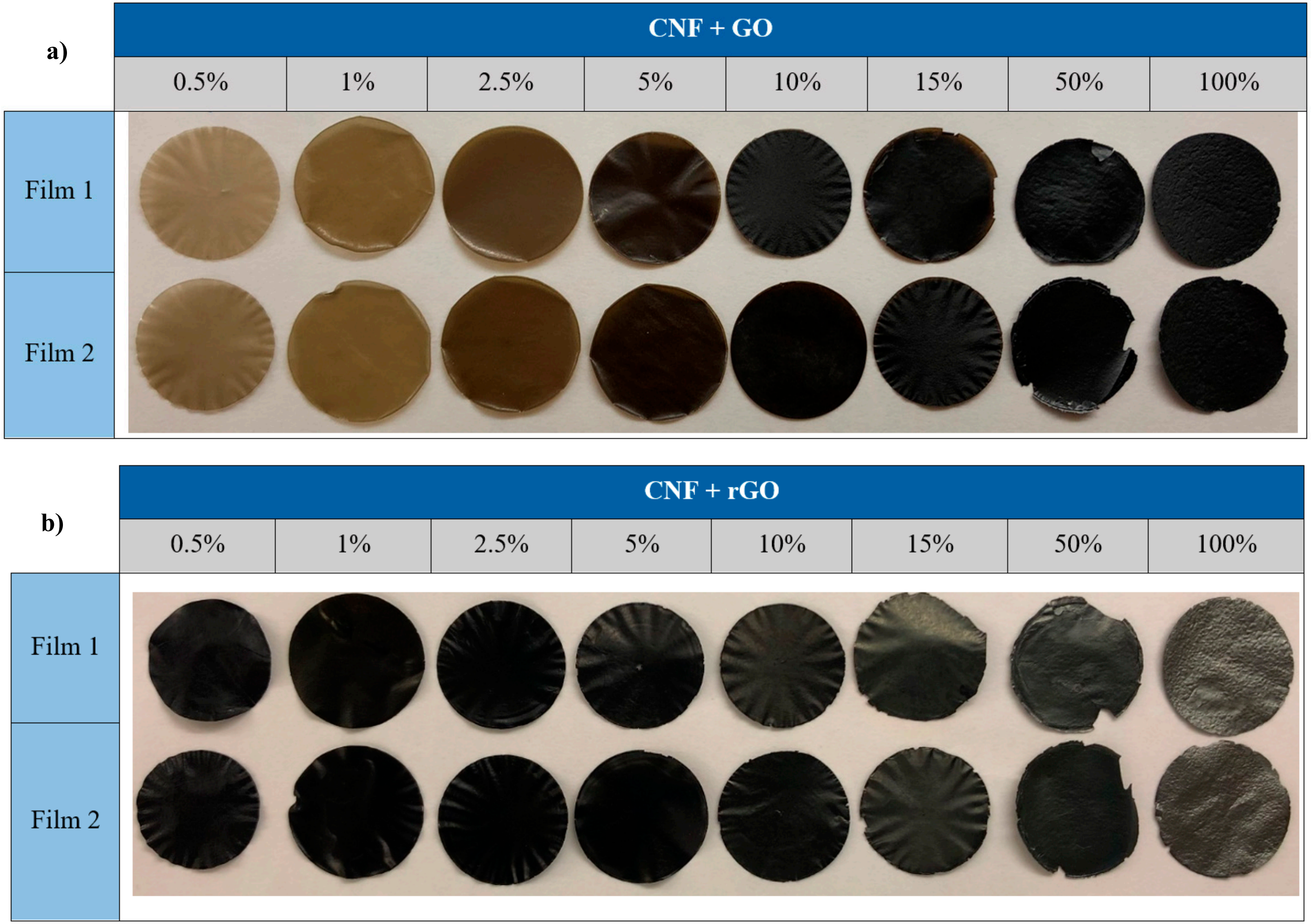

Figure S3. Pictures of the CNF/rGO nanocomposites a) before and b) after the reduction reaction.

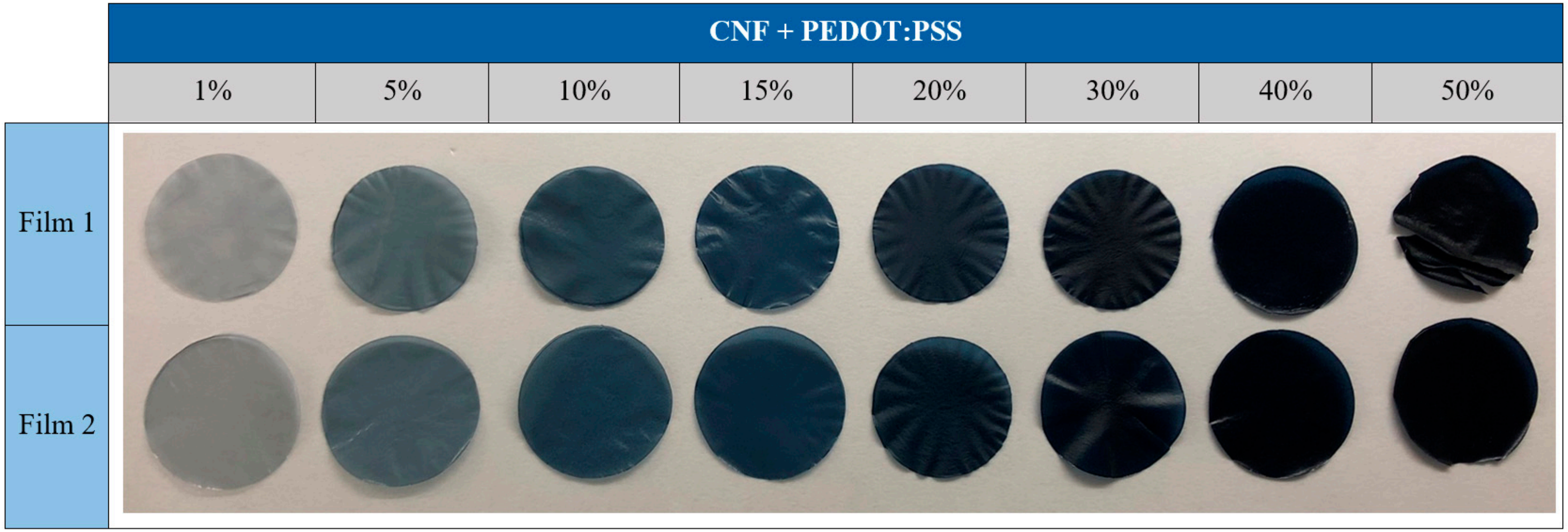

Figure S4. Pictures of the CNF/PEDOT:PSS nanocomposites

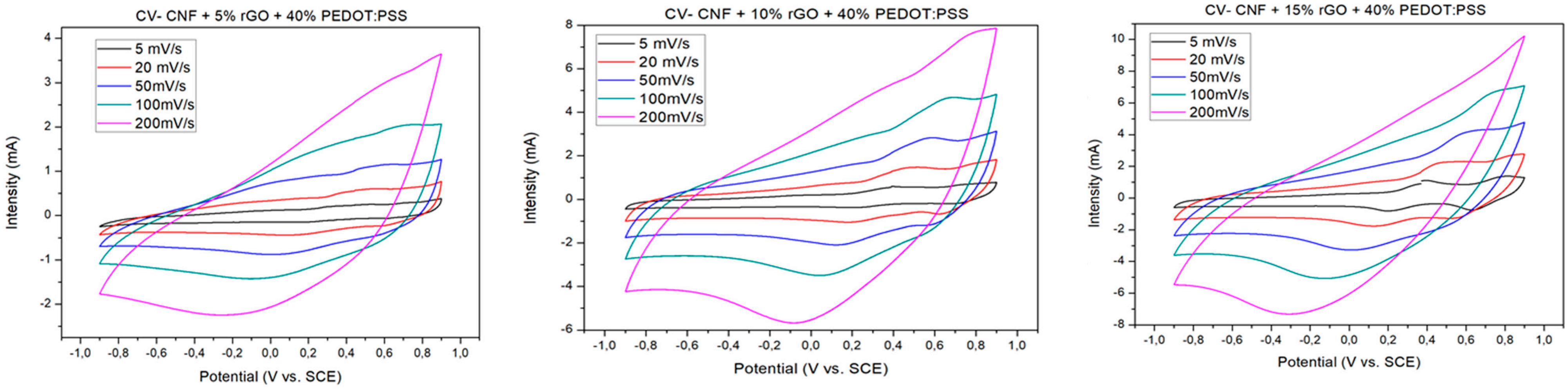

Figure S5. Electrochemical analysis by CV curves of ternary nanocompostes formed by CNF/rGO/PEDOT:PSS under different load (% wt) as energy-storage electrode materials in 2 M NaCl solution at different scan rates.

Table S1. CNF and rGO nanopapers composition.

Experiment	CNF (%)	rGO (%)	Suspension of CNF 1.328 wt% (g)	Suspension of GO 0.4 wt% (g)
1	99.50	0.50	2.99	0.05
2	99.00	1.00	2.98	0.10
3	97.50	2.50	2.93	0.25
4	95.00	5.00	2.86	0.50
5	90.00	10.00	2.71	1.00
6	85.00	15.00	2.56	1.50
7	50.00	50.00	1.50	5.00
8	0.00	100.00	0.00	10.00

All the experiments were performed considerate a theoretical dry weight of nanopaper of 0.040 g.

Table S2. CNF and PEDOT:PSS nanopapers composition

Experiment	CNF (%)	PEDOT:PSS (%)	Suspension of CNF 1.328 wt% (g)	Suspension of PEDOT:PSS 3-4 wt% (g)
1	99.00	1.00	2.98	0.01
2	95.00	5.00	2.86	0.05
3	90.00	10.00	2.71	0.11
4	85.00	15.00	2.56	0.17
5	80.00	20.00	2.41	0.22
6	70.00	30.00	2.10	0.34
7	60.00	40.00	1.80	0.45
8	50.00	50.00	1.50	0.57

All the experiments were performed considerate a theoretical dry weight of nanopaper of 0.040 g.

Table S3. Geometric correction factors

w/s	I/w=1	I/w=2	I/w=3	I/w=4
1			0.2204	0.2205
1.25			0.2751	0.2751
1.5		0.3263	0.3286	0.3286
1.75		0.3794	0.3803	0.3803
2		0.4292	0.4297	0.4297
2.5		0.5192	0.5194	0.5194
3	0.5422	0.5957	0.5958	0.5958
4	0.6870	0.7115	0.7115	0.7115
5	0.7744	0.7887	0.7887	0.7887
7.5	0.8846	0.8905	0.8905	0.8905
10	0.9313	0.9345	0.9345	0.9345
15	0.9682	0.9696	0.9696	0.9696
20	0.9822	0.9830	0.9830	0.9830
40	0.9955	0.9957	0.9957	0.9957
infinit	1	1	1	1

Hence, for samples characterization was considered:

Length (I)=17 mm

Width (w)= 5 mm

Probe spacing (s)= 1.6

Therefore:

I/w= 3.400

w/s= 3.125

Interpolating the correction factor is obtained **C= 0.61035**
